# 

**Published:** 1899-03

**Authors:** 


### *REPRINTS RECEIVED.*

Report of Death, Following Immediately an Operation for Nasopharyngeal Adenoids under Chloroform. By F. W. Hinkel, A.M., M.D.

The Surgical Treatment of Fibroid Tumors of the Uterus; The Technique of Extra Abdominal Shortening of the Round Ligaments; Senile Endometritis and Vaginitis. By Augustin H. Goelet, M.D., New York.

A Case of Concentric Displacement of the Heart to the Right, Presenting Some Unusual Features; A Case of Landry's Paralysis. Both by Chas. Lyman Greene M.D., and J. L. Rothrock, M.D., St. Paul, Minn.

The Treatment of Heart Disease by Saline Baths and Resisted Movements, Schott Method. By Charles Lyman Greene, M.D., St. Paul, Minn.

The Milk Supply of Cities; Can it be Improved. By H. O. Marcy, M.D., LL.D., Boston, Mass.

Cylindrical Transposition. By Norburne B. Jenkins, M.D., Knoxville, Tenn.

Psychiatry in the Southern States. By T. O. Powell, M.D., Milledgeville, Ga., Superintendent of State Lunatic Asylum.

Acute Suppuration of the Middle Ear. By Jas. E. Logan, Kansas City, Mo.

Headache from Nasal Causes. By J. A. Thompson, M.D., Cincinnati, Ohio.

Annual Report of the Wyoming General Hospital.

Medical Education; The Physician in Practice. By Leo M. Crafts, B.L., M.D., Minneapolis.

Modern Possibilities in Chronic Catarrhal Deafness. By Sargent F. Snow, M.D., Syracuse, N. Y.

